# Engineering a Microbial Consortium Based Whole-Cell System for Efficient Production of Glutarate From L-Lysine

**DOI:** 10.3389/fmicb.2019.00341

**Published:** 2019-02-26

**Authors:** Xin Wang, Rui Su, Kequan Chen, Sheng Xu, Jiao Feng, Pingkai Ouyang

**Affiliations:** State Key Laboratory of Materials-Oriented Chemical Engineering, College of Biotechnology and Pharmaceutical Engineering, Nanjing Tech University, Nanjing, China

**Keywords:** glutarate, 5-aminovalerate accumulation, engineering microbial consortium, whole-cell, *E. coli*

## Abstract

Glutarate is an important C5 platform chemical produced during the catabolism of L-lysine through 5-aminovalerate (5-AMV) pathway. Here, we first established a whole-cell biocatalysis system for the glutarate production from L-lysine with the engineered *Escherichia coli* (*E. coli*) that co-expressed *DavAB* and *GabDT*. However, the accumulation of intermediate 5-AMV was identified as one important factor limiting glutarate production. Meanwhile, the negative interaction of co-expressing *DavAB* and *GabDT* in a single cell was also confirmed. Here, we solved these problems through engineering a microbial consortium composed of two engineered *E. coli* strains, BL21-22AB and BL21-YDT, as the whole-cell biocatalysts, each of which contains a part of the glutarate pathway. After the optimization of bioconversion conditions, including temperature, metal ion additives, pH, and cell ratio, 17.2 g/L glutarate was obtained from 20 g/L L-lysine with a yield of 95.1%, which was improved by 19.2% compared with that in a single cell. Little accumulation of 5-AMV was detected. Even at the high substrate concentration, the reduced 5-AMV accumulation and increased glutarate production were achieved. This synthetic consortium produced 43.8 g/L glutarate via a fed-batch strategy, the highest titer reported to date.

## Introduction

As the seriousness of environmental problems related to global warming and depletion of oil reserves, the development of sustainable bio-process for petroleum-derived chemicals has attracted increased attention in recent years (Tsuge et al., 2016). Dicarboxylic acids, especially aliphatic dicarboxylic acids, such as succinate, glutarate, adipate, pimelate, and suberate, are important building blocks for the synthesis of polymers and polyamides, such as polyurethanes, polyester polyols and polyamides (Okino et al., 2008; Gobin et al., 2015). Nylon is one of the most important polymers, and produced by copolymerization of various dicarboxylic acids with diamines. Glutarate, as an important C5 building block, has been widely used for the production of nylon, such as nylon-4,5 (a co-polymer of putrescine and glutarate) and nylon-5,5 (a co-polymer of cadaverine and glutarate) (Williams et al., 2003; Adkins et al., 2013; Baldwin et al., 2015; Zhao et al., 2016). For the chemical synthesis of glutarate, a mixture of cyclohexanone, and cyclohexanol (KA oils) was oxidized with nitric acid (Paris et al., 2003; Vafaeezadeh and Hashemi, 2016). However, the cost and environmental concern of chemical methods limited the sustainable production of glutarate in industry. Hence, the efficient bio-based process for glutarate production is highly desired.

A natural pathway for producing glutarate by the four-step degradation of lysine has been identified in *Pseudomonas putida* (*P. putida*) (Adkins et al., 2013; Park et al., 2013; Liu et al., 2014; Rohles et al., 2016), which was also called the 5-aminovalerate (5-AMV) pathway. In this pathway, L-lysine was oxidized to generate 5-aminovaleramide by L-lysine monooxygenase (encoded by *DavB)*, which is then converted to 5-AMV by 5-aminovaleramide amidohydrolase (encoded by *DavA*). Thenceforth, 5-AMV is converted into glutarate via glutarate semialdehyde by aminovalerate aminotransferase (encoded by *GabT*) and succinate semialdehyde dehydrogenase (encoded by *GabD*). The conversion of 5-AMV into glutarate semialdehyde employs α-ketoglutarate (α-KG) as the acceptor for amine group. However, glutarate was not found in the natural metabolites of *P. putida*. Based on this pathway, the highest glutarate titer of 1.7 g/L has been achieved in recombinant *E. coli* (Park et al., 2013). In recent years, several other artificial glutarate biosynthetic pathways were also developed. For example, by incorporation of a “+1” carbon chain extension pathway from α-KG in combination with α-keto acid decarboxylation, 0.42 g/L of glutarate was successfully obtained in the recombinant *E. coli* (Wang et al., 2017). Yu et al. also constructed a novel glutarate biosynthetic pathway from α-KG reduction by expressing eight genes in E. coli, and 3.8 mg/L of glutarate was finally produced (Yu et al., 2017). However, the glutarate production via the *de novo* biosynthesis strategy from glucose remained far below that required for economic applicability due to the long fermentation period and low conversion rate. Thus, it would be desirable to find other alternative method for the biotechnological production of glutarate.

We have developed an efficient whole-cell biocatalysis process for high-level conversion of L-lysine into 5-AMV with a final titer of 240.7 g/L previously (Wang et al., 2016). It is well known that fermentation of high concentrations of L-lysine is easily carried out, and annual production of L-lysine reached more than two million tons currently (Eggeling and Bott, 2015). As the fastest growing segment within the amino acid market, L-lysine is readily available at a reasonable price, about 1500$ per ton. With L-lysine as a starting material, a variety of valuable chemicals have been produced, such as cadaverine (Ma et al., 2015; Oh et al., 2015), pipecolic acid (Ying et al., 2015), and 5-AMV (Adkins et al., 2013; Park et al., 2013, 2014; Wang et al., 2016). As glutarate has a price of about 9500$ per ton, considering these factors, producing glutarate by bioconversion of lysine based on a whole-cell biocatalytic process seems feasible.

In this study, a recombinant *E. coli* was constructed to produce glutarate by the conversion of L-lysine through the overexpression of *DavB*, *DavA*, *GabT*, and *GabD* involved in 5-AMV pathway. When the whole-cell biocatalysis was performed by *E. coli* BL21-*DavAB*-*GabDT*, the accumulation of intermediate 5-AMV was detected and largely restricted glutarate productivity. Furthermore, we confirmed that the co-expression of *DavAB* and *GabDT* in a single cell negatively affected the activity each other. Several metabolic engineering strategies have been employed to solve the intermediate accumulation problems, such as increasing the expression strength of the downstream gene by means of promoter libraries (Miksch et al., 2005), mRNA stability (Smolke et al., 2001), or ribosome binding strength (Salis et al., 2009), and dynamic control of pathway enzymes using intermediate-sensitive promoters (Dahl et al., 2013). Among these methods, the synthetic microbial consortium has attracted researchers’ attention in recent years for the production of valuable metabolites (Lu et al., 2014; Chen and Nielsen, 2016; Yang et al., 2018). As a result, a novel microbial consortia based whole-cell system was engineered by expression of *DavAB* and *GabDT* in two divided *E. coli* strains for glutarate production. The benefits and features of microbial consortia were subsequently characterized with the highest glutarate titer reported to date.

## Materials and Methods

### Microorganisms and Media

All microbial strains employed in this study are listed in Table 1. *E. coli* strains were cultured at 37°C in Luria–Bertani medium (10 g/L peptone, 5 g/L yeast extract, and 5 g/L sodium chloride) and antibiotics were added at the following concentrations: 100 mg/L ampicillin (Amp), 35 mg/L chloramphenicol (Cm), and 50 mg/L kanamycin (Kan).

**Table 1:** Strains and plasmids used in this study.

Strains or plasmids	Description	References
***E. coli* strains**		
BL21(DE3)	Used as host strain	Invitrogen
BL21-22AB	*E. coli* BL21(DE3) harboring plasmid pET22b-DavAB	This study
BL21-YDT	*E. coli* BL21(DE3) harboring plasmid pACYC-GabDT	This study
BL21-22AB-YDT	*E. coli* BL21(DE3) harboring plasmid pET22b-DavAB and pACYC-GabDT	This study
**Plasmids**		
pET22b	expression vector, Amp^R^, P_T7_, pBR322 ori	This study
pACYCDuet-1	expression vector, Cm^R^, P_T7_,P15A ori	This study
pACYC-GabDT	Gene *GabD* inserted between *Nco*I and *Hin*dIII sites of pACYCDuet-1 and *GabT* inserted between *Nde*I and *Xho*I sites of pACYCDuet-1	This study
pET22b-DavAB	Gene *DavA* inserted between *Age*I and *Xma*I sites of pET22b and *DavB* inserted between *Nde*I and *Xho*I sites of pET22b	This study

### Plasmids Construction

All plasmids used or constructed in this study were listed in Table 1. pET22b-*DavAB* was constructed by sequential insertion of synthesized *DavB* and *DavA* genes from *Pseudomonas putida* (*P. putida*) KT2440 into pET22b at the *Nde*I/*Xho*I and *Age*I/*Xma*I sites, respectively. pACYC-*GabDT* was constructed by sequentially cloning the *GabT* and *GabD* genes from *P. putid*a KT2440 into pACYCDuet-1 at the *Nde*I/*Xho*I and *Nco*I/*Hind*III sites, respectively. Expression of all genes was confirmed by sodium dodecyl sulfate polyacrylamide gel electrophoresis (SDS–PAGE) analysis.

### *DavAB* and *GabDT* Activities *in vivo*

*DavAB* and *GabDT*, expressed in *E. coli* with the plasmid pET22b-*DavAB* and pACYC-*GabDT*, were subjected to enzyme activity assays. The *DavAB* assay mix contained resting cells BL21-22AB-YDT (OD_600_ = 5) and 5 g/L L-lysine in 50 mM PBS buffer (pH 7.0) in a total volume of 10 mL. Reactions were performed at 37°C for 3 h. Samples were removed and determined by high-performance liquid chromatography (HPLC) (described below). One unit (U) of *DavAB* activity was defined as 1 μmol 5-AMV produced per min under the described conditions. For calculating specific units (U/mg protein), the protein concentration was estimated based on the OD_600_ of the culture and the assumption that 1 L culture with an OD_600_ of 1 contains 0.250 g of biomass of which half is assumed to be protein.

The *GabDT* assay mix contained resting cells BL21-22AB-YDT (OD_600_ = 5), 12 g/L α-KG, and 5 g/L 5-AMV in 50 mM PBS buffer (pH 7.0) in a total volume of 10 mL. Reactions were performed at 37°C for 3 h. Samples were removed and determined by HPLC (described below). One unit (U) of *GabDT* activity was defined as 1 μmol glutarate produced per min under the described conditions. For calculating specific units (U/mg protein), the protein concentration was estimated based on the OD_600_ of the culture and the assumption that 1 L culture with an OD_600_ of 1 contains 0.250 g of biomass of which half is assumed to be protein.

### Preparation and Optimization of the Whole-Cell Biocatalysts

Recombinant strain was inoculated into 5 mL LB medium with desirable antibiotics from a freshly transformed single colony at 37°C for 8 h. Then the seed culture was transferred into 100 mL LB medium in baffled Erlenmeyer flasks with an initial optical density (OD_600_) of 0.05. Cell densities were detected by UV spectrophotometry at 600 nm. When OD_600_ reached 0.3, 0.6, 1.0, or 2.0 approximately, the cultures were induced by 0.1, 0.25, 0.5, or 1.0 mM IPTG at 15, 20, 25, 30, or 37°C, respectively, to optimize the gene expression. After 12 h of incubation, cells were harvested by centrifugation at 4000 × *g* for 10 min, discarding the supernatant and washing twice in cold 50 mM PBS buffer (pH 7.0). Finally, the cells were re-suspended in PBS buffer as the whole-cell biocatalysts.

### Recombinant Whole-Cell Bioconversion for Glutarate Production

The whole-cell bioconversion was carried out in a 50 mL flask with 20 mL reaction broth containing resting cells BL21-22AB-YDT (OD_600_ = 20), adding different concentrations of L-lysine and α-KG with the molar ratio of 1:1 and the rest were complemented with 50 mM PBS buffer (pH 7.0). The added content of L-lysine was 10, 20, 30, 60, and 80 g/L, respectively, α-KG the same. The pH value was controlled at about 7.0 with phosphoric acid and sodium hydroxide solutions. The reaction was performed at the temperature of 37°C and stirred at 200 rpm. Samples were taken at the specific intervals to measure the concentrations of glutarate, 5-AMV, L-lysine, and α-KG.

In this section, the mutual effect of *DavAB* and *GabDT* activities based on the expression in a one cell was explored. The resting cells, including BL21-22AB-YDT, BL21-22AB, and BL21-YDT were prepared as biocatalysts. To test the effect of *DavAB* expression on *GabDT* activity, the whole-cell transformation of 5-AMV to glutarate was carried out in a 20 mL reaction mixture containing 30 g/L α-KG, 15 g/L 5-AMV, and 50 mM PBS buffer (pH = 7.0) in a 50 mL flask with resting cells BL21-22AB-YDT (OD_600_ = 20). As a contrast, the activity of the whole-cell BL21-YDT was determined at the same reaction conditions. To test the effect of *GabDT* expression on *DavAB* activity, the transformation of L-lysine to 5-AMV was carried out in a 50 mL flask containing 20 g/L L-lysine with resting cells of BL21-22AB-YDT (OD_600_ = 20), while the whole-cell BL21-22AB was used as the control. All the reaction was performed at the temperature of 37°C and stimulated with a paddle speed of 200 rpm in a thermostatic shaker. Samples were taken at the specific intervals to measure the concentrations of substrates, products and intermediates.

### The Engineered Microbial Consortia for Whole-Cell Bioconversion

The engineered microbial consortia including BL21-22AB (OD_600_ = 10) and BL21-YDT (OD_600_ = 10) was employed for whole-cell bioconversion of L-lysine to glutarate in the presence of 20 g/L α-KG and 20 g/L L-lysine. The reaction was also performed with 20 OD_600_ of BL21-22AB-YDT and 10 OD_600_ of BL21-22AB-YDT, respectively, as the control. The temperature was maintained at 37°C and stimulated with a paddle speed of 200 rpm in a 1 L fermenter. Samples were taken at the specific intervals to measure the concentration of substrates, products, and intermediates.

To enhance glutarate yield, the effects of pH, reaction temperature, metal ion, and cell ratio were investigated. The effect of temperature was determined by measuring the whole-cell conversion activity between 20 and 55°C and the pH were between 5 and 8.2. To determine the metal ions preference, the effect of Zn^2+^, Fe^3+^, Fe^2+^, K^+^, Mg^2+^, Co^2+^, Mn^2+^, Sr^2+^, Ca^2+^, and Cu^2+^ (3 mM) additives was investigated. The proportion of BL21-22AB and BL21-YDT was adjusted to measure the reduction of intermediate products.

Fed-batch bioconversion was carried out in a 1 L fermenter with a total reaction mixture volume of 300 mL. The recombinant strain was cultured and induced as described above. The reaction mixture containing resting cells BL21-22AB (OD_600_ = 10) and BL21-YDT (OD_600_ = 10), 20 g/L L-lysine, 5 mmol/L Cu^2+^, and 0.5% Triton X-100 was incubated at 37°C with a speed of 200 rpm. Samples were taken at the specific intervals to measure L-lysine concentration. L-lysine powder was added into the reactor to maintain the substrate concentration at 20 g/L, when the L-lysine decreased to 1–2 g/L. Samples were taken at the specific intervals until the reactions reached equilibrium.

### Analytical Methods

Cell growth was monitored by absorbance at OD_600_ by ultraviolet spectrophotometer. The quantity analysis of substrates and intermediates was performed by liquid chromatography. All samples were measured by two methods. The first method is the quantity of glutarate and α-KG which was performed by a HPLC system (Agilent 1260 series, Santa Clara, CA, United States) equipped with a UV-Vis detector and a Bio-Rad Aminex HPX-87H column (300 mm^∗^7.8 mm). The column temperature was maintained at 55°C and the mobile phase was 0.8 mM H_2_SO_4_ at a flow rate of 0.6 mL/min. To analyze the aqueous concentrations of L-lysine and 5-AMV, a HPLC (Agilent 1100 series, Santa Clara, CA, United States) system was used, which was equipped with an evaporative light scattering detector (ELSD) and a Prevail C18 column (250^∗^4.6 mm, 5 μm, Bio-Rad, Hercules, CA, United States) at 28.5°C. 0.7% (v/v) trifluoroacetic acid aqueous solution was used as the mobile phase at a flow rate of 1.0 mL/min. The product yield was calculated as follows: Glutarate yield (%) = C(G)/C(L), where C(L) represents the initial L-lysine molar concentration, and C(G) represents the increased glutarate molar concentration after reaction.

## Results

### The Co-expression of *DavAB* and *GabDT* in a Single Cell to Produce Glutarate

**FIGURE 1:**
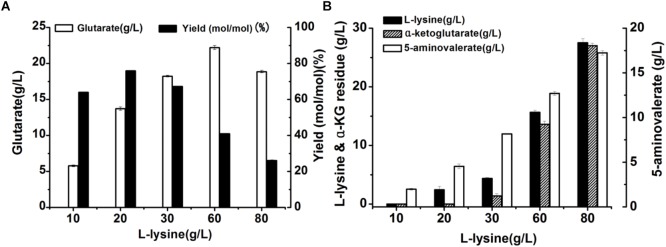
Recombinant whole-cell bioconversion for glutarate production via co-expression of *DavAB* and *GabDT* in *E. coli* BL21(DE3). **(A)** The glutarate production under the different initial concentrations of L-lysine after bioconversion of 70 h; **(B)** The L-lysine and α-ketoglutarate (α-KG) residue, and 5-AMV accumulation in the whole-cell bioconversion system under the different initial concentrations of L-lysine after bioconversion of 70 h.

Here, we attempted to develop a whole-cell biocatalytic system for glutarate production from L-lysine. Accordingly, a recombinant *E. coli* BL21-22AB-YDT was constructed by co-expressing four genes of *DavAB* and *GabDT* in *E. coli* BL21(DE3) as the whole-cell catalysts. The SDS-PAGE illustrated that the protein *DavA*, *DavB*, *GabD*, and *GabT* mainly existed in the supernatant, indicating a soluble expression (Supplementary Figure S1 and Supplementary Table S1). Subsequently, the bioconversion was carried out with the different concentrations of L-lysine ranging from 10 to 80 g/L. As shown in Figure 1A, the glutarate production improved with the increasing L-lysine concentration, and reached the highest level (22.2 g/L) at 60 g/L L-lysine. Furthermore, we found that the highest glutarate molar yield was only 75.9% under the condition of 20 g/L L-lysine. When the L-lysine concentration increased to 60 g/L, a lower molar yield of 41% was obtained, which was decreased to 26.1% at 80 g/L lysine.

To explore the potential reasons involved in low yield of glutarate, the metabolite distribution in the bioconversion mixture was tested. As shown in Figure 1B, the intermediate 5-AMV was largely accumulated no matter what the initial concentration of L-lysine was. Under the condition of 20 g/L L-lysine, 5-AMV accumulation from L-lysine was 4.6 g/L, while 17.2 g/L 5-AMV was accumulated under the condition of 80 g/L lysine. The 5-AMV accumulation reflected a metabolic balance in the recombinant strain *E. coli* BL21-22AB-YDT. To confirm it, the *in vivo* activity of *DavAB* and *GabDT* was measured. As a result, the *in vivo* activity of *DavAB* was 1.46 U/mg and the *in vivo* activity of *GabDT* was 0.3 U/mg, indicating an imbalance of the *DavAB* and *GabDT* activities when they co-expressed in the recombinant strain BL21-22AB-YDT.

**FIGURE 2:**
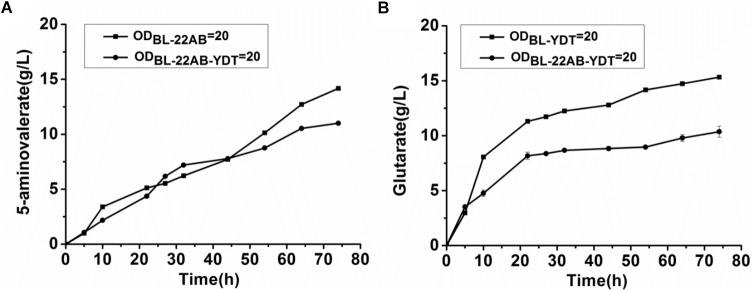
The negative interactions of co-expressing *DavAB* and *GabDT* in *E. coli* BL21(DE3). **(A)** The influence of expressing *GabDT* on *DavAB* activity. The *in vivo DavAB* activity in strain BL21-22AB was compared to that in the strain BL21-22AB-YDT; **(B)** The influence of expressing *DavAB* on *GabDT* activity. The *in vivo GabDT* activity in strain BL21-YDT was compared to that in the strain BL21-22AB-YDT.

**FIGURE 3:**
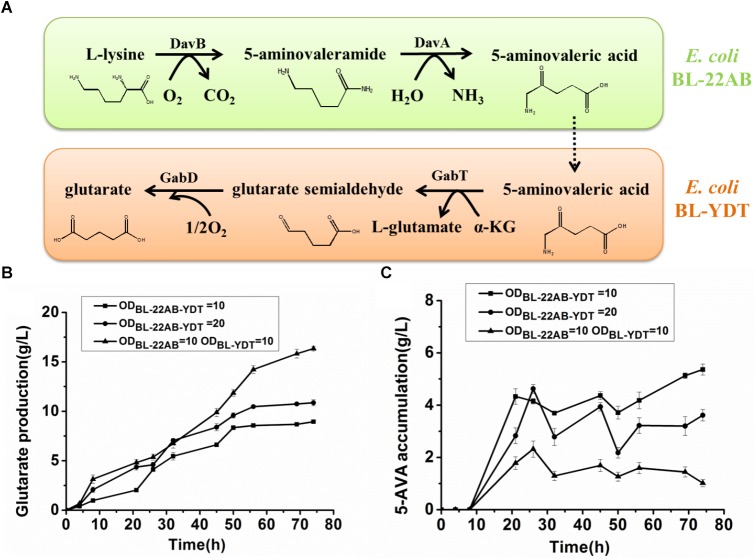
Engineering a synthetic microbial consortium based whole-cell system for production of glutarate from L-lysine. **(A)** A schematic of the synthetic microbial consortium for glutarate production; **(B)** Glutarate production in the synthetic microbial consortium; **(C)** 5-AMV accumulation in the synthetic microbial consortium.

We attempted to enhance the expression of *GabDT* to decrease 5-AMV accumulation by optimization of plasmid copy numbers. However, the increased glutarate production was not found due to the formation of insoluble *GabT* protein (data not shown). Engineering a microbial consortium might be an alternative way to reduce the metabolic imbalance. We first comparatively analyzed the effect on activities of *DavAB* and *GabDT* when expressed in a single strain or two strains. The *DavAB* activity difference in *E. coli* BL21-22AB-YDT and *E. coli* BL21-22AB was shown in Figure 2A. 5-AMV production by the whole cells of *E. coli* BL21-22AB-YDT was lower than that by *E. coli* BL21-22AB, indicating a decreased *DavAB* activity due to the expression of *GabDT*. Meanwhile, a lower *GabDT* activity of *E. coli* BL21-22AB-YDT was also identified compared to that of *E. coli* BL21-YDT through the detection of glutarate production from 5-AMV to α-KG (Figure 2B). These results displayed that the co-expression of *DavAB* and *GabDT* in one cell would have a negative interaction. Considering these results, we established a microbial consortium based whole-cell system composed of *E. coli* BL21-22AB and *E. coli* BL21-YDT (Figure 3A) to eliminate the activity imbalance of *DavAB* and *GabDT*, as well as the interaction of gene expression in a single cell.

### Engineering a Microbial Consortium for Glutarate Production via the Whole-Cell Process

The performance of the microbial consortium based whole-cell system was first evaluated by the determination of 5-AMV accumulation and glutarate production, while the whole-cell BL21-22AB-YDT was performed as the control (Figure 3B,C). For the microbial consortium based whole-cell system, the *E. coli* BL21-22AB and *E. coli* BL21-YDT were added into the bioconversion mixture with an OD_600_ of 10, respectively. As the results shown in Figure 3B, 16.3 g/L glutarate was obtained from 20 g/L L-lysine with a molar yield of 90.2% after bioconversion of 70 h. For the 5-AMV variance in the microbial consortium based whole-cell system, it accumulated to 2.3 g/L at the beginning of 30 h. After that, the 5-AMV concentration gradually reduced to 1.01 g/L at 74 h (Figure 3C). For the control group, 8.9 g/L of glutarate was produced after bioconversion of 74 h with a yield of 49.2%, and 5.4 g/L 5-AMV was accumulated when *E. coli* BL21-22AB-YDT was used as the whole-cell biocatalysts with an OD_600_ of 10 (Figure 3B,C). With an OD_600_ of 20, the glutarate titer was 10.9 g/L, and 3.6 g/L 5-AMV was accumulated finally (Figure 3B,C). Obviously, the microbial consortium based whole-cell system could reduce the intermediate accumulation and enhance glutarate yield efficiently.

### Optimizing Conditions of the Microbial Consortium Based Whole-Cell System

**FIGURE 4:**
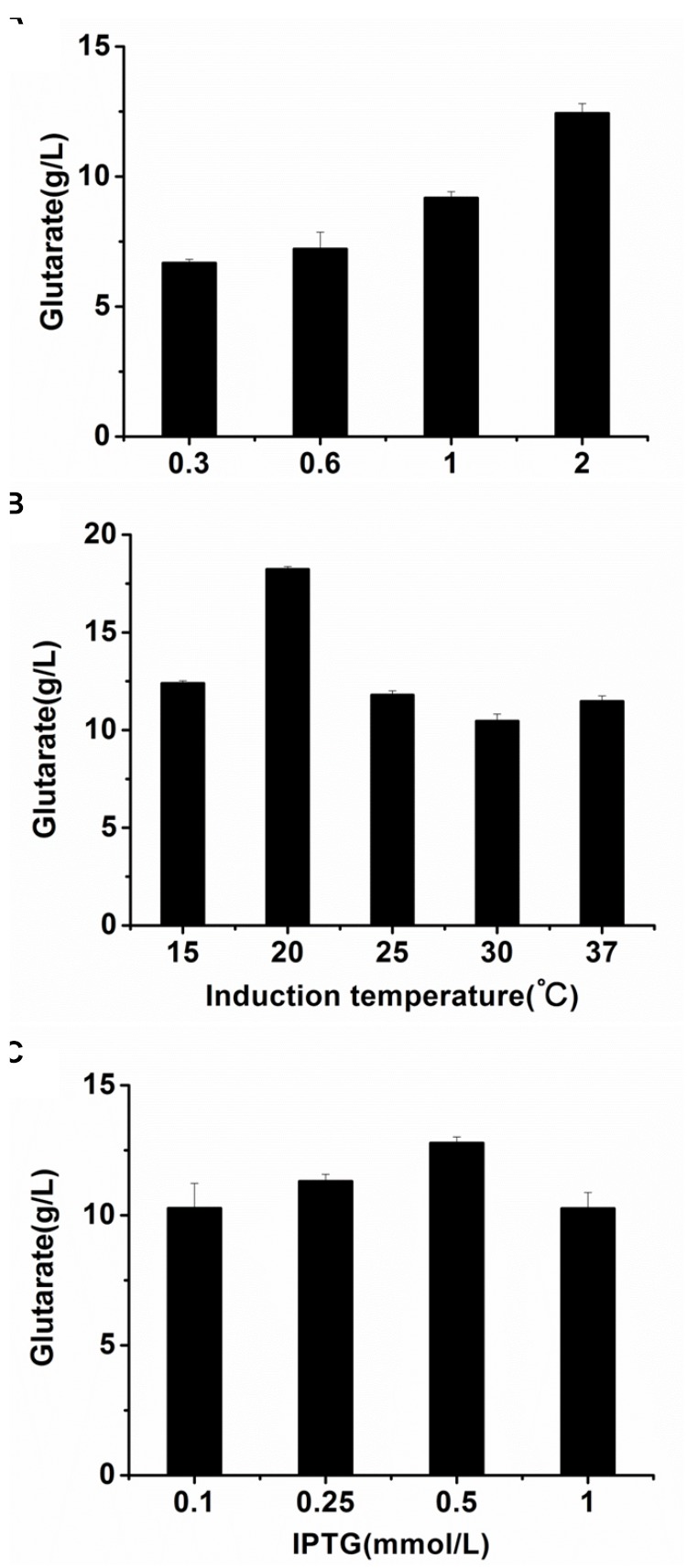
Optimizing the cultivation conditions of whole-cell BL21-YDT. **(A)** Induction time; **(B)** Cultivation temperature; **(C)** IPTG concentration.

The optimal cultivation conditions of *E. coli* BL21-22AB have been identified in the previous study (Wang et al., 2016). To make the whole-cell system more efficient, the cultivation conditions of *E. coli* BL21-YDT including induction time, temperature, and IPTG concentration were optimized. As shown in Figure 4A,B, the optimal induction time and temperature of BL21-YDT were observed during the stationary phase (OD_600_ = 2.0) at 20°C. Additionally, titration of various IPTG concentrations ranging from 0.1 to 1 mM allowed assessment of glutarate production, which peaked at the IPTG concentration of 0.5 mM (Figure 4C).

**FIGURE 5:**
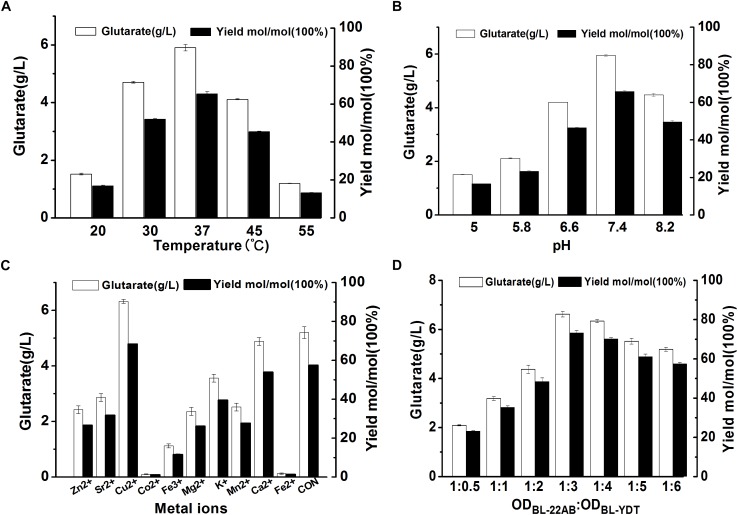
Optimizing the bioconversion conditions of the microbial consortium based whole-cell system. **(A)** The effect of reaction temperature on glutarate production; **(B)** The effect of reaction pH on glutarate production; **(C)** The effect of metal ions on glutarate production; **(D)** The effect of cell ratio on glutarate production.

To further improve biocatalytic efficiency, the bioconversion conditions, including reaction temperature, reaction pH, metal ion additive, and cell ratio were also optimized. The effect of reaction temperature on glutarate production was shown in Figure 5A. Following bioconversion at 20, 30, 37, 45, or 55°C for 30 h, we observed optimal glutarate production at 37°C. The effect of reaction pH on glutarate production was shown in Figure 5B. The results indicated that glutarate production achieved the highest level when reaction pH was at 7.4. The effects of metal ions on glutarate production in the microbial consortium based whole-cell system were shown in Figure 5C. The results indicated that the addition of Cu^2+^ exhibited positive effects with a 1.2-fold higher glutarate production. The addition of Ca^2+^ did not affect whole-cell catalytic activity, whereas the addition of Zn^2+^, Fe^3+^, Fe^2+^, K^+^, Mg^2+^, Co^2+^, Mn^2+^, or Sr^2+^ exhibited negative effects. The presence of Co^2+^ and Fe^2+^ drastically decreased the whole-cell catalytic activity (Figure 5C).

As the accumulation of 5-AMV, we inferred that the activity of *GabDT* might be critical for glutarate productivity. One of the advantages for the microbial consortium is that the cell ratio could be easily adjusted. Thus, we attempted to increase the ratio of BL21-YDT in the whole-cell system to evaluate its effect on glutarate production (Figure 5D). Increasing the ratio of BL21-YDT in the whole cell system improved glutarate production to some extent. When the ratio of BL21-22AB and BL21-YDT was below 1:3, glutarate production was poor with a molar yield lower than 50%. Obviously, when the ratio of BL21-22AB and BL21-YDT was set as 1:3, glutarate production achieved the highest level (Figure 5D). However, the production presented slowly descend tendency with the increasing ratio of BL21-YDT continuously. Thus the appropriate ratio of two strains was important for the glutarate productivity in our microbial consortium based whole-cell system.

### The Production of Glutarate From L-Lysine via the Microbial Consortium Based Whole-Cell System

**FIGURE 6:**
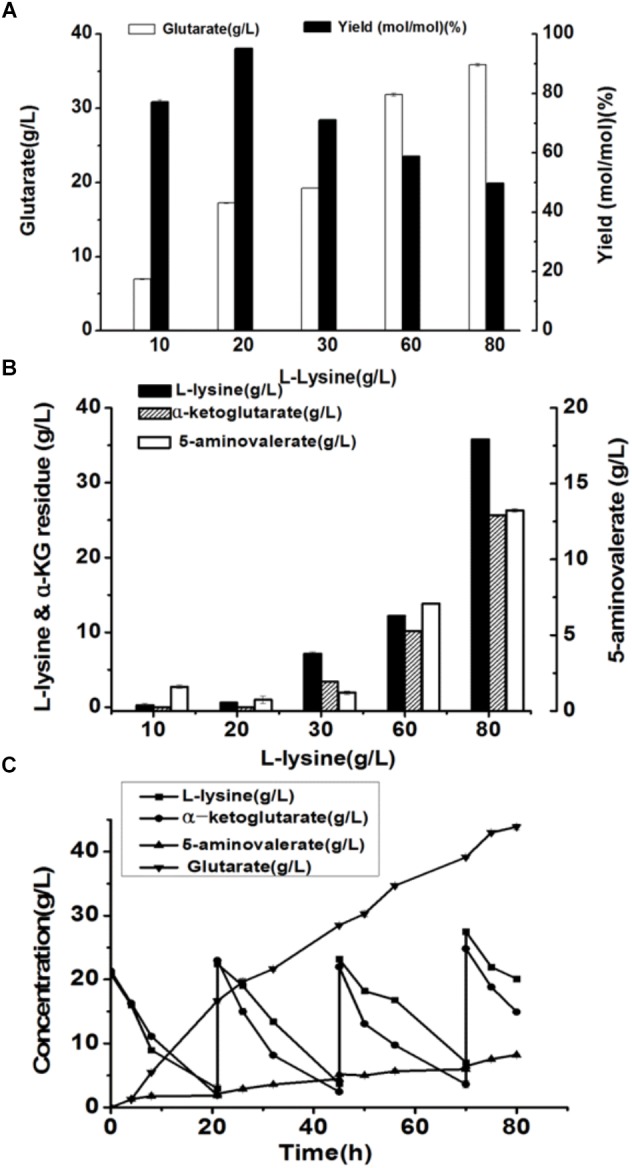
The production of glutarate from L-lysine via the microbial consortium based whole-cell system. **(A)** The glutarate production from the different concentrations of L-lysine with the microbial consortium based whole-cell system after bioconversion of 70 h; **(B)** The L-lysine and α-KG residue, and 5-AMV accumulation with the microbial consortium based whole-cell system after bioconversion of 70 h; **(C)** A fed-batch strategy for glutarate production of from L-lysine with the microbial consortium based whole-cell system. The L-lysine consumption, α-KG consumption, 5-AMV accumulation, and glutarate production during the fed-batch bioconversion were detected.

Under the optimal conditions, glutarate production from L-lysine using the *E. coli* BL21-22AB and *E. coli* BL21-YDT coupling whole-cell system was performed with the different concentrations of L-lysine ranking from 10 to 80 g/L. As shown in Figure 6A, the glutarate titer was increased with the increasing L-lysine concentration. However, we found the glutarate yield achieved the highest level of 95.1% under the condition of 20 g/L L-lysine, and then gradually reduced when the L-lysine concentration increased from 30 to 80 g/L (Figure 6A). Compared to the single strain based whole-cell system, the glutarate production and yield were all significantly improved (Table 2). For example, the highest glutarate yield in the single strain based whole-cell system was 75.9% at a lysine concentration of 20 g/L.

**Table 2:** The performance comparison of the microbial consortium based whole-cell system with that of single strain based whole-cell system.

Initial L-lysine concentration (g/L)	5- aminovalerate accumulation (g/L)		Glutarate production (g/L)		Glutarate molar yied (%)	
	*E. coli* BL21-22AB-YDT	*E. coli* BL21-22AB and *E. coli* BL21-YDT	*E. coli* BL21-22AB-YDT	*E. coli* BL21-22AB and *E. coli* BL21-YDT	*E. coli* BL21-22AB-YDT	*E. coli* BL21-22AB and *E. coli* BL21-YDT
10	1.98	1.59	5.79	6.97^∗∗^	64.02	77.10^∗∗^
20	4.55	0.74^∗∗^	13.73	17.24^∗∗^	75.90	95.15^∗∗∗^
30	8.18	1.21^∗∗∗^	18.23	19.23^∗∗^	67.21	70.95^∗∗^
60	12.71	7.09^∗∗∗^	22.22	31.86^∗∗∗^	40.97	58.76^∗∗∗^
80	17.23	13.23^∗∗^	18.87	35.88^∗∗∗^	26.10	49.63^∗∗∗^

*Significance levels of students t-test, ^∗^P < 0.05, ^∗∗^P < 0.01, ^∗∗∗^P < 0.001 for the performance of microbial consortium compared to the single strain.*

The accumulation of 5-AMV was also monitored. As the results in Figure 6B, little 5-AMV could be detected when the lysine concentration was lower than 30 g/L in our microbial consortium based whole-cell system. Even under a higher lysine concentration of 80 g/L, the accumulation of 5-AMV was reduced to 13.23, which is 17.23 g/L in the single strain based whole-cell system (Table 2). The largely reduced 5-AMV accumulation and increased glutarate production indicated that engineering microbial consortium, each of which contains the part of the pathway, was an efficient strategy to reduce the pathway imbalance for the improvement of glutarate productivity.

Finally, a fed-batch strategy to maintain L-lysine concentration at 20 g/L was performed in a 1 L fermenter. The result was shown in Figure 6C. 16.7 g/L glutarate was produced after bioconversion of 21 h with an average productivity of 0.79 g/L/h. Meanwhile, lysine, and α-KG were almost exhausted and the substrates were added into the bioconversion mixture. We observed that the glutarate production increased to 28.5 g/L from 21 to 45 h with an average productivity of 0.49 g/L/h. In the third batch from 45 to 70 h, glutarate titer was increased to 39.1 g/L with an average productivity of 0.42 g/L/h. After that, the substrate consumption rate decreased to a relatively low level, and no obvious increase in glutarate synthesis was observed with the addition of L-lysine. Finally 43.8 g/L glutarate was obtained after bioconversion of 80 h, which was the highest value reported to date.

## Discussion

In recent years, several C3 and C4 chemicals have been successfully produced in recombinant microorganisms as the potential platform chemicals for synthesis of other value-added chemicals (Zhang et al., 2017). Glutarate is an attractive C5 building block for the production nylon-5,5 and other C5-derived polyesters from renewable feedstocks (Adkins et al., 2013; Park et al., 2014). Here, we developed a whole-cell bioconversion system with recombinant *E. coli* strains for the production of glutarate from L-lysine.

With the recombinant *E. coli* that co-expressed four genes (*DavB*, *DavA*, *GabD*, and *GabT*) involved in 5-AMV pathway as the whole-cell catalysts, the highest titer of 22.2 g/L glutarate was obtained from 60 g/L L-lysine with a yield of 41%. We further identified that the intermediate 5-AMV was largely accumulated, which might be one of important factors limiting glutarate production. Previous studies have indicated that imbalances within metabolic pathways could lead to the accumulation of intermediate metabolites in multiple gene pathways (Xu et al., 2013). The imbalance of *DavAB* and *GabDT* activities in *E. coli* BL21-22BA-YDT was also confirmed here. In addition, when the four genes were co-expressed in one strain, the decreased *DavAB* and *GabDT* activities are both observed. Moreover, the *GabDT* activity was more negatively affected by the expression of *DavAB* compared to the effect of *GabDT* expression on *DavAB* activity. Overexpression of multiple genes in one recombinant host cell often utilizes a significant amount of the host cell’s resources, removing those resources away from host cell metabolism and placing a metabolic load (metabolic drain, metabolic burden) on the cell to support a highly expressed foreign pathway (Glick, 1995). The biochemistry and physiology of the host may change significantly due to the imposed metabolic load, resulting in deleterious outcomes for the recombinant protein (Cao et al., 2016; Horga et al., 2018). The co-expression of *DavAB* and *GabDT* in one strain might overload the host’s capacity and further affected their expression resulting in decreased activity. As *DavAB* was expressed on a high copy number plasmid (pET22b) and *GabDT* on a low copy number plasmid (pACYCDuet-1), the *GabDT*, which was disadvantage in quantity, might be affected more significantly.

In recent years, the synthetic microbial consortium has attracted researchers’ attention as an alternative strategy to reduce the metabolic imbalance for the synthesis value-added chemicals (Brenner et al., 2008). In addition, this design will simplify the optimization of each reaction in the pathway, so that the time required for making the product would be substantially reduced (Brenner et al., 2008). Several reports have successfully achieved the production of biofuels and chemicals by the synthetic consortia comprising genetically engineered microbes (Bayer et al., 2009; Xia et al., 2012; Minty et al., 2013). Therefore, we attempted to develop a synthetic microbial consortia system through expression of *DavAB* and *GabDT* in two divided *E. coli* strains for glutarate production. Exactingly, this synthetic microbial consortia based whole-cell system effectively reduced the accumulation of intermediate 5-AMV, and the glutarate yield could reach 95.1% under the optimal L-lysine concentration. With a fed-batch strategy, a final glutarate titer of 43.8 g/L was achieved.

However, there are still some limitations in our bioconversion system. For example, during the production of 5-AMV, 120 g/L L-lysine could be consumed within 5 h (Wang et al., 2016). However, L-lysine could not be totally exhausted after bioconversion of 70 h even under the condition of 30 g/L L-lysine as shown in our results. We inferred that the production of glutarate might have an inhibition on the substrate conversion, which will be focused on in our following study for the further improvement of glutarate production.

## Author Contributions

XW and RS designed the experiments, generated all strains for this work, performed the bioconversion experiments, prepared the figures, and contributed to the writing of the manuscript. SX and JF helped draft the manuscript. KC conceived the study, directed the experiments, and helped draft the manuscript. PO contributed ideas to the project. All authors contributed to the analysis and interpretation of data. All authors approved the manuscript submitted.

## Conflict of Interest Statement

The authors declare that the research was conducted in the absence of any commercial or financial relationships that could be construed as a potential conflict of interest.

## Supplementary Material

FIGURE S1 SDS-PAGE analysis of the cell-free extract of the recombinant strain BL-22AB, and BL-YDT and BL-22AB-YDT. All the lanes were loaded with an equal amount of protein.

TABLE S1 Evaluating the performance of the microbial consortium based whole-cell system.
