# Supplementary figures and images for: Engineering a Microbial Consortium Based Whole-Cell System for Efficient Production of Glutarate From L-Lysine
Source: Front Microbiol. 2019 Feb 26;10:341. doi: 10.3389/fmicb.2019.00341 (PMC6400078; doi:10.3389/fmicb.2019.00341)

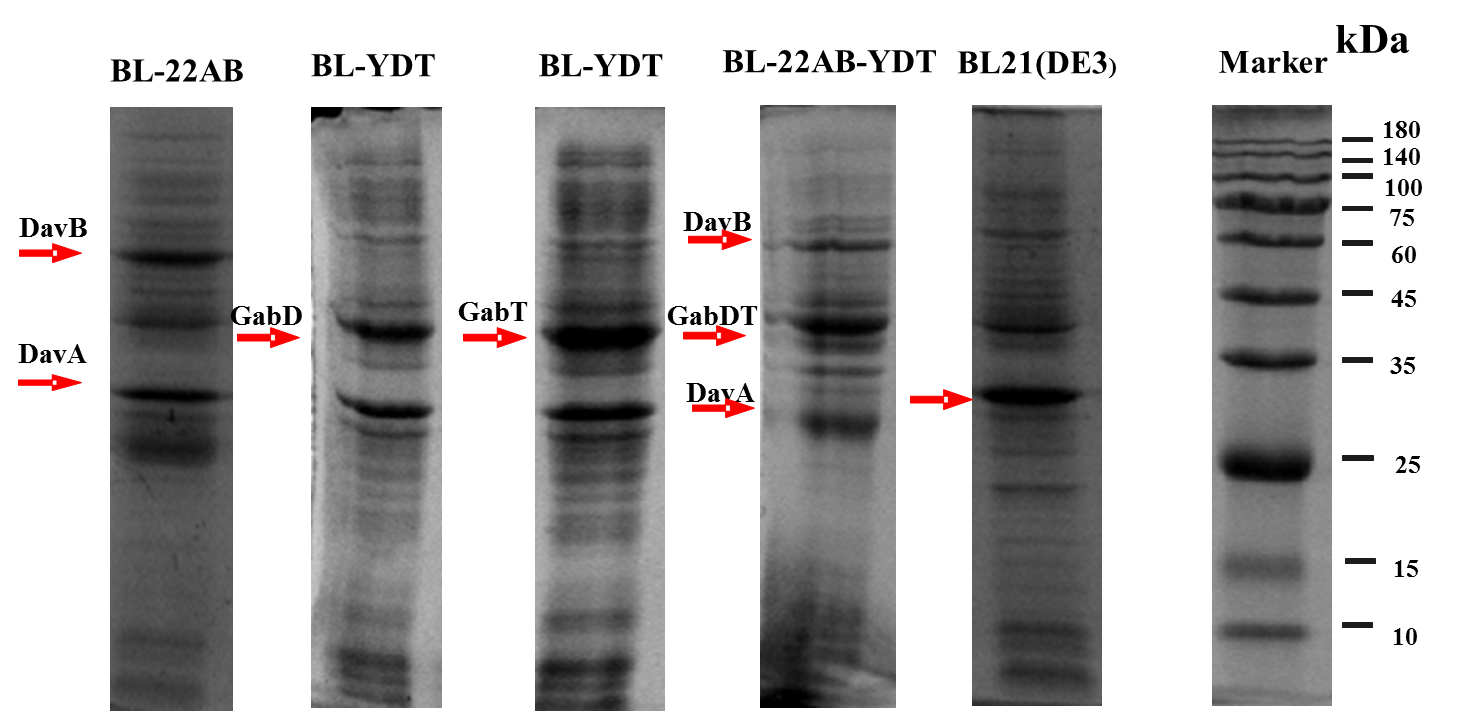

Supplement: FIGURE S1 — SDS-PAGE analysis of the cell-free extract of the recombinant strain BL-22AB, and BL-YDT and BL-22AB-YDT. All the lanes were loaded with an equal amount of protein. [file Image_1.TIF]
