# Supplementary material for: Engineering a Microbial Consortium Based Whole-Cell System for Efficient Production of Glutarate From L-Lysine
Source: Front Microbiol. 2019 Feb 26;10:341. doi: 10.3389/fmicb.2019.00341 (PMC6400078; doi:10.3389/fmicb.2019.00341)
Supplement: TABLE S1 — Evaluating the performance of the microbial consortium based whole-cell system. [file Table_1.docx]

**Supplementary Table S1** Evaluating the performance of the microbial consortium based whole-cell system.

| cell | OD_600_ | 5-aminovalerate accumulation (g/L) | Glutarate production (g/L) |
| --- | --- | --- | --- |
| *E.coli* BL-22AB-YDT | 10 | 5.36 | 8.95 |
| *E.coli* BL21-22AB-YDT | 20 | 3.61 | 10.87 |
| *E.coli* BL21-22AB  and  *E.coli* BL21-YDT | 10 and 10 | 1.02 | 16.35 |
